# Auxin and cytokinin control fate determination of cotyledons in the one-leaf plant *Monophyllaea glabra*

**DOI:** 10.3389/fpls.2022.980138

**Published:** 2022-09-02

**Authors:** Ayaka Kinoshita, Hirokazu Tsukaya

**Affiliations:** Graduate School of Science, The University of Tokyo, Tokyo, Japan

**Keywords:** auxin, cytokinin, fate determination, indeterminate growth, one-leaf plant, *Monophyllaea glabra*

## Abstract

One-leaf plants in the Gesneriaceae family initially have two cotyledons of identical size; one cotyledon stops growing shortly after germination, whereas the other continues indeterminate growth. Factors involved in the unequal growth have been investigated, and a competitive relationship between the two cotyledons was previously proposed. However, questions regarding the fate determination of the two cotyledons remain: Why does only one cotyledon grow indeterminately while the other stops; is the fate of the cotyledons reversible; and what role does light quality play in the fate determination of the cotyledons? In this study, physiological experiments using the one-leaf plant species *Monophyllaea glabra* suggest that a biased auxin concentration between the two cotyledons and subsequent cytokinin levels may determine the fate of the cotyledons. In addition, observation of relatively mature individuals without hormone treatment and younger individuals with cytokinin treatment under laboratory growth conditions revealed that the fate determination of the microcotyledon is reversible. Although light quality has been suggested to be important for the determination of cotyledon fate in *Streptocarpus rexii*, an anisocotylous species, we conclude that light quality is not important in *M. glabra*.

## Introduction

Typical seed plants produce a shoot (composed of stems, leaves, and axial buds) with indeterminate growth from the shoot apical meristem (SAM). However, lateral organs, such as leaves, show determinate development. One-leaf plants in Gesneriaceae, which include all members of the genus *Monophyllaea* and some members of the genus *Streptocarpus*, have contrasting development compared to typical plants: One-leaf plants do not produce new leaves or stems after germination; instead, one of the cotyledons grows indeterminately.

Notably, one-leaf plants have two cotyledons of identical size just after germination as typical eudicots do, called the isocotylous stage. However, after initial development, one of the cotyledons (the microcotyledon) stops growing, whereas the other cotyledon (the macrocotyledon) continues to grow; this is called the anisocotylous stage. Although such indeterminate growth of the cotyledon is an extreme case represented by one-leaf plants, unequal growth of the two cotyledons is observed among plants in the Didymocarpoideae in Gesneriaceae, to which both the known one-leaf plants genera belong. The other subfamilies in Gesneriaceae do not show the unequal growth between two cotyledons. The unequal growth of two cotyledons, called anisocotyly (Fritsch, 1904), in one-leaf plants has attracted scientific interest for more than 100 years (Crocker, 1860; Chifflot, 1909).

To interpret the unique development of plants in the Gesneriaceae, including one-leaf plants, a concept called the phyllomorph, which is thought to correspond to the shoot (Kinoshita and Tsukaya, 2018), was introduced (Jong, 1970; Jong and Burtt, 1975). A phyllomorph is composed of an indeterminately growing leaf and a petiole-like structure. Basically, a one-leaf plant is composed of only one phyllomorph derived from a macrocotyledon, a cotyledonary phyllomorph. In *Streptocarpus*, there is a rosulate species, *S. rexii*, composed of multiple phyllomorphs including a macrocotyledon, arranged in an irregular rosette. Although this species is not a one-leaf plant, the growth of the macrocotyledon and newly produced leaves of phyllomorphs persist for long time.

There are three kinds of unique meristems in the basal part of the macrocotyledon, the groove meristem (GM), the basal meristem (BM), and the petiolode meristem, which are thought to contribute to the development of the inflorescence, lamina expansion, and elongation of the petiole-like stalk (petiolode), respectively (Jong, 1970; Jong and Burtt, 1975). The GM and the BM are thought to correspond to the SAM and leaf meristem of typical seed plants, respectively, based on studies of histological characters and gene expression (Jong and Burtt, 1975; Imaichi et al., 2000, 2001; Ayano et al., 2005; Ishikawa et al., 2017; Kinoshita et al., 2020). However, the GM is different from the SAM in that it does not produce new organs in the vegetative phase; the BM is different from the leaf meristem in that the cell proliferative activity is indeterminate.

Regarding the timing of the cotyledon fate determination, Oehlkers (1923) suggested that the two cotyledons have the same physiological features just after germination. This hypothesis was supported by Tsukaya (1997), who removed the cotyledons of *Monophyllaea horsfieldii* just after germination and observed that the remaining cotyledon differentiated into the macrocotyledon. From this result, Tsukaya (1997) proposed that the two cotyledons compete with each other, similar to apical dominance in which removal of the apical shoot induces the growth of lateral shoots. It has also been reported that some one-leaf plants in the genus *Streptocarpus* and *Monophyllaea*, both cotyledons occasionally grow continuously (Burtt, 1978; Tsukaya, 1997), again suggesting that the cotyledons have the same potential, at least just after germination.

Tsukaya (1997) also revealed that the likelihood of growth of the remaining cotyledon after the other is removed decreases as the timing of the removal is delayed, suggesting that the fate of the cotyledons becomes gradually irreversible as development progresses. However, substantial growth of the microcotyledons under greenhouse conditions has also been reported (Chifflot, 1909), although they remain smaller than macrocotyledons, suggesting a possibility that microcotyledons can retain a macrocotyledon-like nature.

Some phytohormones induce curious effects in the cotyledon growth of anisocotylous plants. For example, in species in *Monophyllaea* and *Streptocarpus*, the application of exogenous cytokinin to culture medium or soil can cause both cotyledons to grow (Rosenblum and Basile, 1984; Nishii et al., 2004, 2012b; Mantegazza et al., 2007, 2009). Light quality is also known to affect the determination of the cotyledon fate in *S. rexii*, as typical anisocotyly was observed under blue light, whereas the two cotyledons remained small under red light (Saueregger and Weber, 2003; Nishii et al., 2012a). By contrast, red light enhanced anisocotyly of *Microchirita lavandulacea*, an anisocotylous species in Didymocarpoideae (Saueregger and Weber, 2003).

Despite the experiments and observation performed to this time to reveal the nature of anisocotyly, the underlying molecular mechanisms controlling cotyledon fate are still unclear. Furthermore, whether fate determination is reversible or not is controversial. Moreover, whether environmental factors known to affect anisocotyly in some taxa also affect anisocotyly in *Monophyllaea* has not been investigated. In addition it is noteworthy that molecular systems behind the BM activity may differ between the two one-leaf plant genera, because expression pattern of Class I KNOX gene, a key factor for formation and maintenance of SAM, differs between them (Kinoshita et al., 2020).

In this study, to investigate the role of phytohormones in each cotyledon, we used lanolin paste to apply phytohormones to the cotyledons of *M. glabra*, which has been well established as a model one-leaf plant species (Ishikawa et al., 2017; Kinoshita and Tsukaya, 2018; Kinoshita et al., 2020). Our results suggest that the cotyledon with a higher auxin concentration becomes the microcotyledon, whereas a certain cytokinin concentration induces growth of the macrocotyledon. In addition, observation of relatively mature individuals without phytohormone treatment and younger individuals with cytokinin treatment in the anisocotylous stage, both under our laboratory conditions, showed that the fate determination of the microcotyledon is reversible. Moreover, both red and blue light could induce anisocotyly in *M. glabra* as they did in *Microchirita lavandulacea*, an anisocotylous Gesneriaceae species, although previous research showed that anisocotyly was suppressed under red light in *S. rexii*, suggesting that the quality of light might not be a critical factor in fate determination of anisocotylous species in Gesneriaceae in general.

## Results

### Both cotyledons have the same potential to become macrocotyledons just after germination in *Monophyllaea glabra*

**FIGURE 1:**
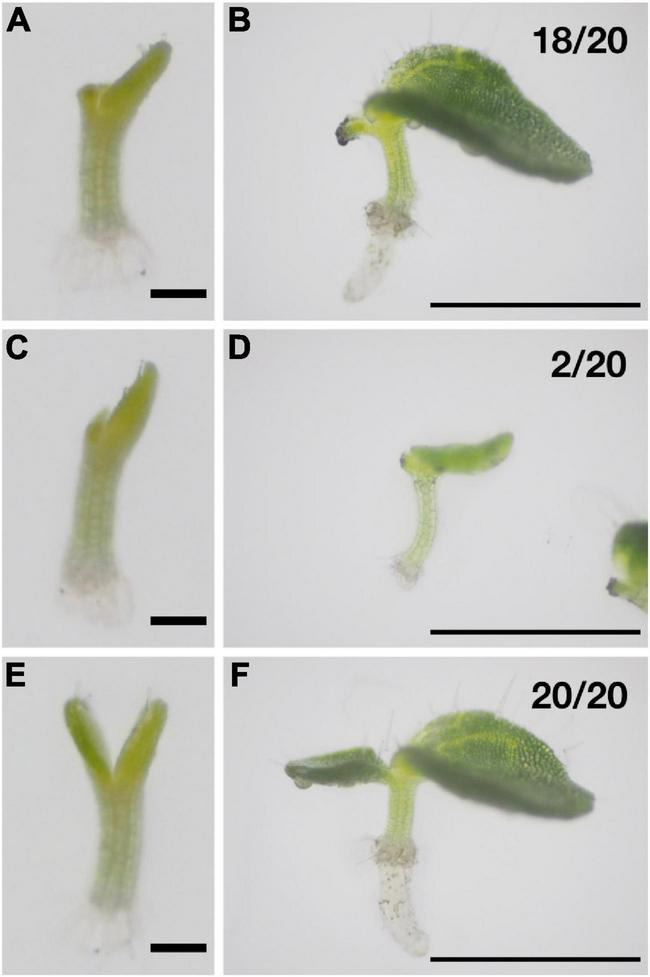
Both of the cotyledons have the same potential to become a macrocotyledon at the early isocotylous stage in *M. glabra*. **(A, B)** One of the cotyledons was removed at 6 DAS **(A)**, and the remaining cotyledon became the macrocotyledon by 10 days after cutting (DAC) **(B)**. **(C,D)** One of the cotyledons was removed at 6 DAS **(C)**, and the remaining cotyledon became the microcotyledon by 10 DAC **(D)**. **(E,F)** A control sample. 6 DAS **(E)** and 16 DAS **(F)**. Bar = 1 mm in **(A,C,E)** and 1 cm in **(B,D,F)**.

In *M. horsfieldii*, when one of the cotyledons was removed in the early isocotylous stage, the other cotyledon survived and became the macrocotyledon (Tsukaya, 1997), suggesting that both cotyledons of *M. horsfieldii* initially have the potential to grow indeterminately. We conducted a similar experiment using *M. glabra* to test whether this nature was conserved among species in the genus *Monophyllaea*. We removed one of the cotyledons just after (within 24 h) unfolding of cotyledons (Figures 1A,C). Intact individuals were also prepared as controls (Figure 1E). Ten days after cutting, the remaining cotyledon grew as a macrocotyledon in 18 out of 20 samples (Figure 1B), suggesting that both cotyledons initially had the same potential to become macrocotyledons, consistent with the result of Tsukaya (1997). The remaining cotyledon did not grow in the other two samples (Figure 1D). In all the control individuals, one of the cotyledons grew larger than the other and became the macrocotyledon (Figure 1F). These results strongly suggest that the fate of the two cotyledons is variable just after germination in *M. glabra*; a similar result was obtained in *M. horsfieldii* (Tsukaya, 1997); therefore, it is likely that this plastic character of the cotyledons in the isocotylous stage just after germination is conserved at least in the genus *Monophyllaea*.

### Auxin suppresses—and cytokinin promotes—the growth of cotyledons when applied to cotyledons in the isocotylous stage

**FIGURE 2:**
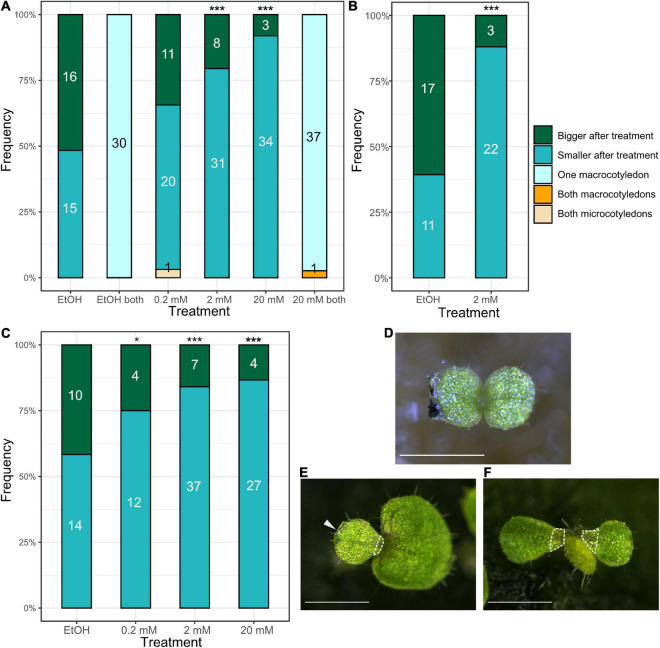
Auxin treatment of cotyledons in the isocotylous stage reveals that a higher concentration in one of the cotyledons induces the microcotyledon fate. **(A–C)** The results of three independent auxin application experiments. “Bigger after treatment” and “Smaller after treatment” indicate individuals in which the cotyledon treated with the lanolin paste grew larger (macrocotyledon) and those that stopped growth (microcotyledon) after treatment, respectively. “One macrocotyledon” indicates that one of the cotyledons differentiated into a macrocotyledon and the other differentiated into a microcotyledon. “Both macrocotyledons” and “Both microcotyledons” indicate individuals that harbored two macrocotyledons and two microcotyledons, respectively. The numbers in the bars indicate the absolute number of individuals in each category. The x-axis shows the types of treatment: “EtOH” indicates the treatment with lanolin only containing ethanol, used to dilute IAAK. “0.2 mM,” “2 mM,” and “20 mM” indicate the concentration of auxin dissolved in the lanolin in the treatment. On the x axis, “both” indicates that the lanolin was applied to both of the cotyledons. **(D)** The stage at which the lanolin treatment was conducted. Lanolin was applied to the edge of the individual cotyledon, and black carbon particles were stuck on the lanolin to make it easier to show the position of the lanolin. **(E)** One of the individuals treated with 20 mM IAAK, observed in experiment **(A)**. The arrow indicates the lanolin, which can be seen on the cotyledon on the left (microcotyledon). The hatched white line shows narrower part in the microcotyledon lamina. **(F)** The individual harboring both microcotyledons in experiment **(A)**. A new organ was produced between the two cotyledons. The hatched white line shows narrower part in the microcotyledon lamina. *p < 0.05, ***p < 0.001 in a binomial test of “Bigger after treatment” and “Smaller after treatment” (The number of occurrences of “Both macrocotyledons” and “Both microcotyledons” is not included because they are very rare). Bar = 0.5 mm in **(D)** and 1 mm in **(E,F)**.

To test whether the bias of the concentration of phytohormones between the two cotyledons affects their fate, we used lanolin, an oil from wool, to apply phytohormones to a single cotyledon; this method of exogenous phytohormone application is widely used in plant research (e.g., Watahiki and Yamamoto, 1997; Reinhardt et al., 2000; Scarpella et al., 2006). In a previous study, the authors applied phytohormones to each cotyledon on a species of *Streptocarpus* using aquaphore, whose main component is also oil. However, the application of aquaphore to both cotyledons inhibited the growth of both cotyledons (Rosenblum and Basile, 1984). Therefore, it was necessary to improve the experimental system. To prevent harmful effects as much as possible from both physical and physiological aspects, the lanolin paste treatments were applied to the edge of the cotyledon but not on the top surface of the cotyledon (Figure 2D). When the lanolin, containing ethanol (EtOH), used in the cytokinin experiments as a solvent and here as a control, was applied to only one cotyledon, it seemed to have no effect on its fate, as statistically significant difference was not detected in the number between micro- and macrocotyledons were formed from the treated cotyledons (Figures 2A–C; EtOH). Furthermore, when lanolin was applied to both cotyledons, one of the cotyledons became larger than the other cotyledon in all the individuals, similar to the non-treated plants (Figure 2A; EtOH both). These results suggest that the fate determination of the cotyledons was not affected by lanolin application in the experimental system. Here, we judged the differentiation of macro- and microcotyledons not only by the size of the leaf lamina but also by the presence of a petiole-like narrow part at the proximal end, which is made by elongation of differentiate cells and the absence of further cell proliferation (Figures 2E,F).

The effect of auxin on the fate determination of the cotyledons was investigated using this lanolin paste experimental system. Lanolin paste containing different concentrations of auxin [indole-3-acetic acid potassium salt (IAAK); 0.2, 2, and 20 mM] was applied to one of the cotyledons. The auxin-treated cotyledons differentiated into microcotyledons more frequently than into macrocotyledons, and this tendency was more obvious when the auxin concentration was higher (Figures 2A–C). Strikingly, when 2 and 20 mM IAAK was used, 31 out of 39 (79%) and 34 out of 37 individuals (92%) with the auxin-treated cotyledons, respectively, differentiated into microcotyledons (Figure 2A); a binomial test indicated that the percentage of the number of cotyledons differentiating into the microcotyledons was significantly higher than at random. We conducted two more independent experiments, and all the experiments showed the same tendency: In a second experiment, 22 out of 25 individuals with the auxin-treated cotyledons differentiated into microcotyledons when 2 mM IAAK was applied (Figure 2B); in the third experiment, 37 out of 44 and 27 out of 31 individuals with the auxin-treated cotyledons differentiated into microcotyledons when they were treated with 2 and 20 mM IAAK, respectively (Figure 2C). Furthermore, when 20 mM IAAK was applied to both cotyledons, one grew as a macrocotyledon and the other grew as a microcotyledon in most of the individuals (37 out of 38 individuals), similar to the non-treated plants (Figure 2A; 20 mM both). Notably, when both cotyledons were treated with 0.2 mM IAAK, we observed an individual in which both cotyledons differentiated into microcotyledons and a phyllomorph-like structure was produced between the two cotyledons (Figure 2F).

**FIGURE 3:**
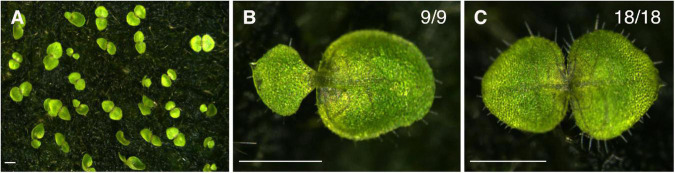
Application of cytokinin to one of the cotyledons in the isocotylous stage induces both cotyledons to grow as macrocotyledons. **(A,C)** Individuals treated with lanolin containing cytokinin. All the individuals (18 out of 18) treated with cytokinin had two cotyledons with lateral veins and a wide leaf lamina in the basal part. **(B)** All the individuals (9 out of 9) treated with lanolin containing DMSO showed clear anisocotyly. Bar = 1 mm.

The effect of cytokinin was also examined in the same manner. We applied 6-benzylaminopurine (BAP), a synthetic cytokinin, at a concentration of 5 mM in lanolin to one of the cotyledons. This resulted in the similar growth of both cotyledons in all individuals (Figures 3A,C). Both cotyledons had lateral veins and a wide leaf lamina in the basal part, which are characteristic of macrocotyledons. By contrast, when lanolin containing only dimethyl sulfoxide (DMSO), a solvent for BAP, was applied, one of the cotyledons remained small and the leaf lamina of the cotyledon became narrower in the basal part due to cell elongation without further cell proliferation, similar to in the non-treated plants (Figure 3B).

These results suggest that a higher concentration of auxin in one cotyledon than the other suppresses the fate of the macrocotyledon and that cytokinin above a threshold level promotes the fate of the macrocotyledon.

### Microcotyledons resume growth without hormone treatment under laboratory conditions

Although *Monophyllaea* species are called one-leaf plants, under certain cultivation conditions, the microcotyledon, which remains small or withers away in wild habitats, grows to some extent (Chifflot, 1909; Oehlkers, 1923). However, it is not known whether the microcotyledon growth continues throughout the life of the plant or if it pauses for a time and then restarts.

**FIGURE 4:**
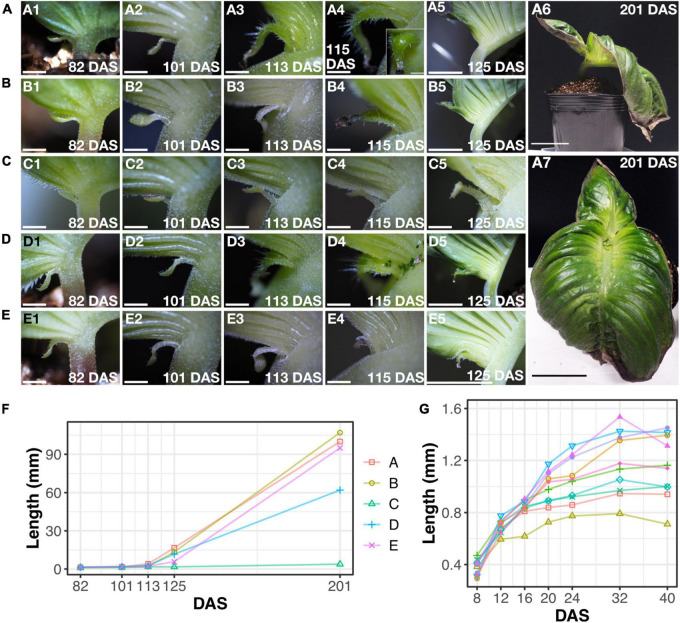
Microcotyledons resume growth under laboratory conditions. **(A–E)** Five individuals, **(A–E)** at 82, 101, 113, 115, 125, and 201 [only for **(A)**] DAS (1–6). The inset in (A4) is a view from the adaxial side of the microcotyledon. (A7) is the frontal view of the individual in (A6). **(F)** The length of the microcotyledons of each individual, **(A–E)**. **(G)** The length of microcotyledons in 10 additional individuals at 8–40 DAS. Bar = 1 mm in (1–4) in **(A–E)** and (C5), 1 cm in (5) in **(A,B,D,E)**, and 5 cm in (A6, A7).

We noticed growth of the microcotyledon in *M. glabra* in our laboratory conditions where we grow *M. glabra* for collecting seeds or other research purposes (continuous light, 22–23°C) (Kinoshita and Tsukaya, 2018; Kinoshita et al., 2020), so we decided to observe the process of the growth carefully. We observed five individuals of *M. glabra* from 82 to 201 days after sowing (DAS) and determined that all the microcotyledons grew to some extent during this time (Figures 4A–E and Table 1). Especially for four individuals at 201 DAS the length of the microcotyledon was more than 50 times longer than the length at 82 DAS (Figure 4A1, A6, A7 and Table 1).

**TABLE 1:** Length of the microcotyledon.

Individual	DAS				
	82	101	113	125	201
A	1.26	1.57	4.03	16.7	100
B	1.50	1.80	1.99	13.4	107
C	1.02	1.18	1.70	1.70	3.79
D	1.47	1.90	2.79	11.7	62.0
E	1.47	1.82	2.24	5.72	95.0

Each individual (A–E) corresponds to the individuals A–E in Figure 4. Length of unit: mm.

Also, at 101 DAS, the length of the microcotyledons was slightly longer than that at 82 DAS (Figures 4A–E and Table 1). The elongation occurred in the petiole-like part of the microcotyledons where the color was whiter (more colorless) than that of the leaf lamina. At 115 DAS, drastic changes in the microcotyledons started to appear in three individuals, (“A”, “B”, and “D” in Figure 4A4, B4, D4). Greener tissues and wider lamina with longer trichomes than the petiole-like part were produced in the basal part of the microcotyledon, leaving the initial microcotyledon lamina in the distal part. This phenomenon also became evident in another individual by 125 DAS (“E” in Figure 4E5). After these drastic changes at 125 DAS, the growth rate increased considerably (Figure 4F).

The drastic changes of microcotyledon growth in the four individuals suggested that this was due to resumed growth after a pause rather than a continuation of growth. To test whether the growth of the microcotyledon stops in earlier stages, we observed 10 other individuals grown under the same conditions and measured the length of the microcotyledons. The length reached a plateau around 40 DAS (Figure 4G) in all individuals. The microcotyledon length was around 0.8 to 1.5 mm, which was almost the same as in the other five individuals at 82 DAS (Table 1), suggesting that growth of microcotyledons almost stopped around 40–80 DAS when grown under the laboratory conditions. In summary, in our laboratory conditions, the microcotyledon of *M. glabra* stops growing for a while, but resumes growth just as macrocotyledons without plant hormone treatment.

### Cytokinin induces the resumption of the microcotyledon growth even when it is applied to microcotyledons in the anisocotylous stage

As mentioned above, we observed a pause in growth followed by resumed growth of microcotyledons in our laboratory conditions. However, the factors that control this restart of growth have yet to be revealed. Since cytokinin can induce both cotyledons to become macrocotyledons when applied in the isocotylous stage, we suspected that exogeneous cytokinin can make fate-determined microcotyledons grow as macrocotyledons.

**FIGURE 5:**
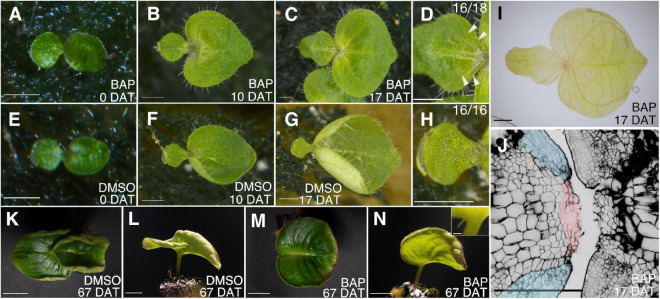
Application of cytokinin to microcotyledons in the anisocotylous stage induces resumption of the growth of microcotyledons. An individual plant treated with lanolin containing cytokinin **(A–D)**; an individual plant treated with lanolin containing DMSO **(E–H)**. 0 days after treatment (DAT) **(A,E)**, 10 DAT **(B,F)**, and 17 DAT **(C,D,G,H)**. The microcotyledons in **(C)** and **(G)** are magnified in **(D)** and **(H)**, respectively. The arrowheads in **(D)** point to lateral veins. **(I)** An individual treated with lanolin containing cytokinin fixed at 17 DAT. **(J)** The outline of epidermal and subepidermal cells in the basal part of macro- and microcotyledons in the same individual as **(I).** The pink and blue shaded areas are the putative GM and BM, respectively, in the microcotyledon resuming growth. **(K–N)** Individuals treated with cytokinin **(K,L)** or DMSO **(M,N)** at 67 DAT. Top views **(K,M)** and side views **(L,N)**. The inset in **(N)** is the magnified view of the microcotyledon of the individual in **(N)**. Bar = 1 mm in **(A–J)**, 1 cm in **(K–N)**, and 100 μm in the inset of **(N)**.

To investigate this, lanolin containing cytokinin (BAP) or DMSO as a control was applied to the microcotyledon of individual plants in the anisocotylous stage (25 DAS, Figures 5A,E). In this stage, the shape of the microcotyledon was petiole-like in the basal part, whereas the basal part of the macrocotyledon was rounded. Ten days after treatment (DAT), at 35 DAS, the basal part of the microcotyledon seemed slightly wider in cytokinin treated individuals than the control, but the difference was not clear (Figures 5B,F). At 17 DAT, obvious microcotyledon growth, which was marked by new lateral veins (clearly observed in 16 out of 18 individuals) and/or longer trichomes were observed in the cytokinin-treated individuals, whereas no such phenotype was observed among all 16 individuals in the control condition (Figures 5C,D,G,H). In a cytokinin-treated sample at 17 DAT, the GM-like and the BM-like tissues, which are composed of small cells, which positioned in the same way as the GM and the BM in macrocotyledon (Imaichi et al., 2001; Ayano et al., 2005) were observed in the basal part of the microcotyledon (Figures 5I,J). Before the resumption of the growth of the microcotyledon, it has been observed in *M. glabra* that cells in the basal part of the microcotyledons are expanded, suggesting they are differentiated (Ayano et al., 2005; Kinoshita et al., 2020). At 62 DAT, in some individuals, microcotyledons grew to the extent that they were difficult to distinguish from macrocotyledons (Figures 5K–N). Therefore, cotyledons seem to retain the potential to grow like a macrocotyledon even after fate-determination as a microcotyledon.

### Anisocotyly can be induced under both red and blue light

The effect of light quality on anisocotyly has been investigated in several Gesneriaceae species. Although all of them belong to the subfamily Didymoracpoideae, their reaction to red or blue light seems to differ among species. In *S. rexii*, typical anisocotyly was induced under blue light, whereas an isocotylous phenotype was observed under red light (Saueregger and Weber, 2003; Nishii et al., 2012a). Contrarily, the anisocotylous phenotype was enhanced in *M. lavandulacea* (Saueregger and Weber, 2003) under red light. Therefore, there is a possibility that the external factors that induce anisocotyly may differ among anisocotylous species. Therefore, we examined the reaction of *M. glabra* to different qualities of light.

Red-light, blue-light, white-light, and dark conditions (see Materials and methods) were examined. For the red- and blue-light treatments, we compared two conditions: Seeds were irradiated with red or blue light just after sowing, or seeds irradiated with white light for 7 days were transferred to red or blue light.

**FIGURE 6:**
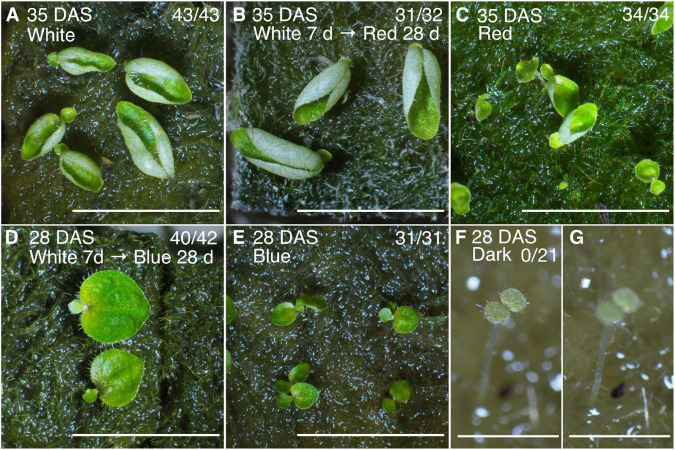
Anisocotylous growth of *M. glabra* is observed both under red and blue light conditions. **(A)** Individuals at 35 DAS grown under white fluorescent light. **(B)** Individuals at 35 DAS grown under white fluorescent light for the first 7 days and red light for the subsequent 28 days. **(C)** Individuals at 28 DAS grown under red light. **(D)** Individuals at 35 DAS grown under white fluorescent light for the first 7 days and blue light for the subsequent 28 days. **(E)** Individuals at 28 DAS grown under blue light. **(F,G)** An individual at 28 DAS grown under dark conditions. The focus is on the cotyledons in **(F)** and on the petiolode in **(G)**. The denominator and numerator at the top right in each figure indicate the number of individuals germinated and the number of anisocotyl individuals, respectively. Bar = 1 cm in **(A–E)** and 1 mm in **(F,G)**.

We observed that anisocotyly was induced among almost all the individuals under all the tested conditions (Figures 6A–E), except for the dark condition (Figure 6F). The individuals grown under red light had macrocotyledons that curled up to the adaxial side (Figure 6C), whereas when grown under blue light, the macrocotyledons grew flat (Figures 6D,E). All individuals germinated under the dark condition showed an isocotylous phenotype with an elongated petiolode (Figures 6F,G). In summary, both red and blue light can induce the anisocotyly in *M. glabra*.

## Discussion

### Cotyledon growth is suppressed by auxin and promoted by cytokinin

In this study, we examined the effects of phytohormones on the fate determination of cotyledons in *M. glabra.* Auxin treatment of one cotyledon resulted in a greater propensity for that cotyledon to differentiate into a microcotyledon, and this tendency increased as the concentration of auxin increased. These results suggest that auxin may have a suppressive role in the growth of the cotyledons in *M. glabra*. Auxin appears to be working in a relative manner between the two cotyledons, rather than in an absolute dose-dependent manner, because application of a high concentration auxin to both did not affect the fate determination (Figure 2A). The difference in the concentration of auxin between two cotyledons might be a molecular background of the proposed “competition” (Tsukaya, 1997).

There were, however, some exceptions. In all three independent experiments, the auxin-treated cotyledon became the macrocotyledon in a few individuals even when the concentration of auxin was considerably high (20 mM). This might be because the fate of these cotyledons in these individuals had already been determined before the treatment; thus, they were not affected by exogenous auxin. Similarly, when one of the cotyledons was removed, even within 24 h after the unfolding of the two cotyledons, the remaining cotyledon in 2 out of 20 individuals did not grow as the macrocotyledon. Therefore, cotyledon fate may be determined at a very early stage; alternatively, these plants may have germinated slightly earlier than the other individuals, and thus they were at a slightly more advanced developmental stage at the time of the auxin treatment.

Although auxin suppressed cotyledon growth when it was applied to only one cotyledon, when both cotyledons were treated it did not cause both cotyledons to become microcotyledons. This suggests that the microcotyledon fate is induced when auxin concentration in the cotyledon is higher than the other cotyledon but not when absolute auxin concentration exceed a certain threshold.

Cytokinin treatment to only one of the cotyledons resulted in the growth of both cotyledons. This may suggest that the growth of a cotyledon occurs when cytokinin levels exceed a certain threshold, and the difference in the concentration between two cotyledons is not important for the fate determination.

**FIGURE 7:**
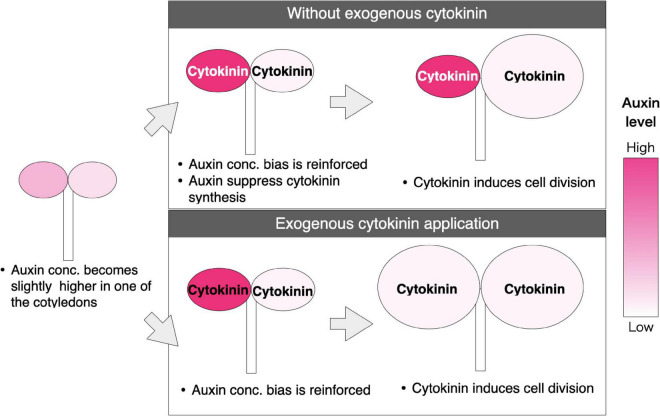
Proposed model for fate determination of the two cotyledons in *M. glabra*. The red color indicates the concentration of auxin. The darker color indicates higher concentration. The black and white color of the letters, “Cytokinin” indicates the high and low concentration of cytokinin in the cotyledons, respectively. The developmental process indicated in the upper part is under a non-treated condition; auxin concentration is slightly different between the two cotyledons in the isocotylous stage. After some time, the difference of the auxin level is enforced due to “up-the-gradient” mechanisms supported by auxin polar transport. Then, cytokinin is produced less in the cotyledon where the auxin level is higher due to the inhibition of cytokinin synthesis by auxin signaling. This results in only one of the cotyledons with a higher cytokinin level becoming a macrocotyledon. The diagram on the bottom shows a developmental process where cytokinin is applied exogenously; when the cytokinin level exceeds a certain threshold in both cotyledons, regardless of the auxin level, both cotyledons can become macrocotyledons.

Apical dominance is achieved by the suppression of cytokinin synthesis by auxin (Tanaka et al., 2006). If the endogenous auxin and cytokinin work in the same way as the exogenous auxin applications in *Monophyllaea*, it is possible that a similar mechanism as that in apical dominance underlies the competitive phenotype of the cotyledons in the following steps: (1) A stochastic difference in the auxin level between two cotyledons is somehow amplified; (2) cytokinin synthesis is then initiated in the cotyledon with a lower auxin level, whereas its production is suppressed in the cotyledon with higher auxin; and 3) cell division in the proximal part is induced in the cotyledon with a higher cytokinin level due to promotion of the expression of cyclin genes by cytokinin signaling (Riou-Khamlichi et al., 1999) (Figure 7). In step 1, auxin transport to the cells with higher auxin levels from the adjacent cells with lower auxin, as explained in the up-the-gradient model (Smith et al., 2006; reviewed in van Berkel et al., 2013), is a possible underlying molecular mechanism for the growth of the macrocotyledon.

### Ability of the microcotyledon to restart growth

Substantial growth of the microcotyledon has been observed in some cultivation conditions in previous studies but has not been observed in wild habitats. In this study, we also observed enhanced growth of the microcotyledons in relatively older individuals under our laboratory growth conditions, and the resumption of growth was induced at a much earlier stage by applying cytokinin to already fate-determined microcotyledons under the same environment.

In our growth conditions, the observation of microcotyledon growth without exogenous application of phytohormones suggested that the growth of the microcotyledon pauses briefly after initial development, but active growth resumes in a later stage. Moreover, we observed the growth of the lamina in the basal part of the microcotyledon. This suggests that cells in the basal part of the microcotyledon retain the potential to become an active leaf meristem or new meristematic cells are provided to the basal part. The substantial growth of microcotyledons has been observed in our laboratory cultivation conditions or in a greenhouse in the previous study (Chifflot, 1909), but not in the wild habitat, so some environmental factors likely affect whether the microcotyledons restart growth. The wild habitat of *Monophyllaea* is often in limestone caves (Burtt, 1978; Kinoshita and Tsukaya, 2018), so it is possible that nutrition and light are more plentiful in the laboratory conditions or in the green house than in the wild habitat, which may promote the unusual development of the microcotyledon.

In the artificially induced growth of the microcotyledon by application of cytokinin, microcotyledons started to produce lamina with long trichomes and lateral veins. Nishii et al. (2004) reported that also in *M. glabra*, the enlarging macrocotyledon in the anisocotylous stage has traits not observed in the isocotylous stage: long trichomes and lateral veins. Therefore, our observations suggest that the growing microcotyledon gained the same identity as the enlarging macrocotyledons. Moreover, the GM- and the BM-like tissues, which comprised small cells, were observed in the basal part of the microcotyledon after the cytokinin treatment, again suggesting that the microcotyledons gained the same identity as the macrocotyledons. Indeed, at 67 DAT, both cotyledons were almost indistinguishable in some individuals. Although Tsukaya (1997) proposed that the fate determination is irreversible by showing that excision of the macrocotyledon did not induce microcotyledon growth, our report of the growth of the microcotyledons suggests that microcotyledons retain the potential for indeterminate growth even after fate determination.

The observation of microcotyledon growth with and without exogenous cytokinin suggests that the restart of the growth of the microcotyledon is related to cytokinin signaling. When microcotyledons resumed growth without exogeneous cytokinin, endogenous cytokinin may be overproduced.

It has been reported that cytokinin promotes cell division in the cotyledons in *Arabidopsis thaliana* (Stoynova-Bakalova et al., 2004), but the role of cytokinin in the establishment of meristematic tissues or indeterminate growth in cotyledons has not been reported. Therefore, the indeterminate growth of the microcotyledon induced by cytokinin is likely to be unique for species in *Monophyllaea* or other species with indeterminately growing leaves. As cytokinin signaling positively regulates class I KNOTTED LIKE HOMEOBOX (KNOX I) gene expression (Rupp et al., 1999; Hamant et al., 2002; Tsuda et al., 2011) and KNOX I promotes cytokinin biosynthesis (Jasinski et al., 2005; Yanai et al., 2005; Sakamoto et al., 2006) resulting in positive feedback, a higher concentration of cytokinin might induce the expression of *SHOOT MERISTEMLESS (STM)*, one of the KNOX I genes, in the basal part of the microcotyledon. If this is true, as *STM* is essential for the formation and maintenance of the SAM (Barton and Poethig, 1993; Endrizzi et al., 1996; Long et al., 1996; Long and Barton, 1998), the corresponding tissue of the SAM in *Monophyllaea*, the GM, may be formed in the microcotyledon in response to cytokinin, leading to the indeterminate growth. Indeed, it has been confirmed that an *STM* ortholog is expressed in the GM of macrocotyledons of *MN. Glabra* (Ishikawa et al., 2017; Kinoshita et al., 2020). The BM may be produced from the GM (Ishikawa et al., 2017), as the leaf meristem is produced from the SAM in typical plants. Therefore, the establishment of the GM may be enough to explain the resumption of indeterminate growth of the microcotyledon.

### Growth of the cotyledon and organ formation

In one-leaf *Streptocarpus* species, it has been observed that new organs are produced between two cotyledons when the growth of both cotyledons is suppressed, such as by application of gibberellic acid, whereas no new organ is produced during the vegetative phase without such a treatment (Nishii et al., 2012b). This phenomenon has been interpreted as a release from apical suppression of the basal meristem (Nishii et al., 2012b).

Although this phenotype has not been induced by gibberellic acid in *Monophyllaea* (Imaichi, 2001), a similar phenomenon was observed in this study when both of the cotyledons were treated with auxin: When the growth of the both cotyledons was suppressed, a new phyllomorph was formed between the two cotyledons. This suggests that, at least in the vegetative phase, the BM also has a suppressive role on the GM in *Monophyllaea* in terms of the formation of new organs. This may also explain the formation of new phyllomorphs after removing the macrocotyledon, although this observation is rare (Tsukaya, 1997).

### Effect of red and blue light on the growth of the cotyledons

We observed that anisocotyly of *M. glabra* was induced under both red- and blue-light conditions, whereas an isocotylous phenotype was observed when grown under dark conditions. Therefore, light is necessary for the anisocotylous development of *M. glabra*. Notably, the reaction to red light differs among species: In *S. rexii*, red light did not induce anisocotyly (Saueregger and Weber, 2003; Nishii et al., 2012a), whereas red light enhanced anisocotyly in *Microchirita lavandulacea* (Saueregger and Weber, 2003). Phylogenetically, *Streptocarpus* and *Microchirita* are more closely related, belonging to the same tribe, Trichosporeae, whereas *Monophyllaea* is relatively distantly related to these genera, belonging to another tribe, Trichosporeae. Therefore, in general, the quality of light might not be important for anisocotyly, but light itself is important. For example, both red and blue light are used for photosynthesis; thus, photosynthetic products, such as sucrose, might be important for anisocotyl growth. Since the intensity and wavelength of the light or whether light receptors are saturated affect the downstream, knocking-out the light-receptors-related genes would be necessary in the future to get solid conclusion on whether light receptors are involved in the determination of the cotyledons.

We also observed that the macrocotyledons curled upward when grown under red light, whereas they grew flat under the blue-light condition. This is consistent with the previous research showing that signaling from the phytochrome, which perceives red light, promotes curling (Kozuka et al., 2013), while the signal from phototropin, which perceives blue light, suppresses curling (Sakamoto and Briggs, 2002; de Carbonnel et al., 2010). However, the direction of curling is opposite in *M. glabra* when compared to *A. thaliana*. It has been suggested in *A. thaliana* that the curling is caused by a lack of cell expansion in the abaxial side (Kozuka et al., 2013). Therefore, *M. glabra* may differ from *A. thaliana* in terms of which side of the epidermal cells reacts to the light.

In conclusion, we have revealed that auxin and cytokinin control the fate determination of cotyledons of the one-leaf plant *Monophyllaea glabra*. In particular, we proposed a new hypothesis that the difference of auxin concentration and subsequent cytokinin concentration between the two cotyledons is important for the competition-like growth of the two cotyledons. Moreover, we demonstrated the ability of the microcotyledon to restart growth as a macrocotyledon. Finally, we revealed that both blue and red light can induce anisocotyly of *M. glabra*. These findings contribute to our understanding of the development of one-leaf plants; furthermore, as one-leaf plants can be regarded as mutants of typical plants with shoots, the elucidation of one-leaf plant development provides general insight into the developmental of typical seed plants.

## Materials and methods

### Plant materials and growth conditions

Seeds of *Monophyllaea glabra* were originally collected at Srakaew Cave, Thailand and have succeeded generation by generation in the laboratory and maintained the strain by collecting seeds in growth chambers (Ishikawa et al., 2017). Seeds were sown on either a block of rockwool or 1/3 MS0 medium adjusted to pH 7.0 by potassium hydroxide with 0.8% (w/v) agar (Wako, Osaka, Japan) depending on experiments. When plants were grown on rockwool for more than 2 months, they were replanted into pots with vermiculite. Plants grown on rockwool or vermiculite were provided with 0.5% (w/w) fine powder hyponex (Hyponex, Osaka, Japan) in tap water. Those plants were grown at 22–23°C under white light provided by fluorescent lamps under continuous-light conditions (Kinoshita and Tsukaya, 2018). The light intensity was ∼45 μmol m^–2^ s^–1^.

### Preparation and application of lanolin paste

Indole acetic acid potassium salt (IAAK; Nacalai Tesque, Kyoto, Japan) was dissolved in Milli-Q water to prepare a 2 M IAAK stock solution. To prepare the lanolin paste containing the final concentrations of 0.2, 2, and 20 mM IAAK, IAAK solutions diluted with 99.5% ethanol were added to lanolin (Wako, Osaka, Japan), heated to 50°C, and vigorously mixed. 6-Benzylaminopurine (BAP; Wako, Osaka, Japan) was dissolved in dimethyl sulfoxide (DMSO; Nacalai Tesque, Kyoto, Japan) to prepare a 500 mM stock solution. Then, 5 mM BAP lanolin paste was prepared by dissolving the stock solution in lanolin. As the controls, we prepared lanolin pastes with only ethanol or DMSO of the same concentration to the corresponding hormone treatments. The lanolin was cooled down to room temperature and picked with an injection needle. A small amount of the lanolin was put on the edge of cotyledons under a dissecting microscope.

### Cultivation under different qualities of light

To test whether the quality of light affects anisocotyly of *M. glabra*, individuals were grown under blue monochromatic light-emitting diode (LED) lights (470 nm) (15–30 μmol s^–2^), red monochromatic LED lights (660 nm) (15–30 μmol s^–2^), white fluorescent lights (15–30 μmol s^–2^), or in darkness. The temperature was kept at 22–23°C. For blue and red light, two conditions were compared: Seeds were kept under the white light condition from just after sowing to 7 DAS and subsequently placed under the blue- or red-light condition; also, seeds were placed under the red- or blue-light condition just after sowing (within 3 h).

### Microscopy

Most of the individuals were observed under a SZ61 dissecting microscope (Olympus, Tokyo, Japan), and the images were taken by an OM-D E-M10 digital camera (Olympus, Tokyo, Japan). For smaller samples, a MZ16 stereomicroscope (Leica Microsystems GmbH, Wetzlar, Germany) was also used. For samples large enough for observation with the naked eye, pictures were taken by a Ricoh WG-4 camera (Ricoh, Tokyo, Japan). To image the basal part of the cells in the microcotyledons in the individuals treated with cytokinin in the anisocotylous stage, the individuals were fixed with 4% PFA with 15% DMSO and 0.1% Tween-20 at 18 days after treatment (DAT). After more than 1 day of the fixation, the sample was immersed in 1% (v/v) calcofluor white stain (Sigma-Aldrich, St. Louis, MO, United States) in ClearSee solution (Kurihara et al., 2015) for more than overnight and observed with a Fluoview FV10i confocal microscope (Olympus, Tokyo, Japan).

## Data availability statement

The original contributions presented in this study are included in the article. Further inquiries can be directed to the corresponding author.

## Author contributions

AK designed the study and performed the experiments. AK and HT directed the study and wrote the manuscript. Both authors contributed to the article and approved the submitted version.
